# Association Between Adolescent Internet Gaming and Adult Problematic Web-Based Board Gaming

**DOI:** 10.3389/fpsyt.2021.591716

**Published:** 2021-04-14

**Authors:** Hanil Ryoo, Sujin Bae, Sun Mi Kim, Kyoung Joon Min, Doug Hyun Han

**Affiliations:** Department of Psychiatry, Chung Ang University Hospital, Seoul, South Korea

**Keywords:** internet gameplay, problematic web board gameplay scale, psychological scales, interaction scales, adolescent

## Abstract

**Introduction:** The results of studies comparing the characteristics of Internet gaming with those of Internet gambling have been controversial. We hypothesized that problematic web-based board gaming behaviors are associated with psychological and social interaction factors. We also hypothesized that non-problematic adolescent Internet gaming is a protective factor against problematic web-based board gaming and that problematic Internet gaming is a predictive factor for problematic web-based board gaming.

**Methods:** We recruited 104 adults who reported engaging in web-based gaming. All participants were asked to complete the Problematic Web Board Gameplay Scale, Center for Epidemiologic Studies Depression Scale (CESD), State-Trait Anxiety Inventory (STAI), Adult Attention Deficit/Hyperactivity Disorder Self-Report Scale (K-AADHD), Family Environmental Scale (FES), Social Avoidance and Distress Scale (SADS), and questionnaires on their web-based board gaming patterns and Internet gaming history.

**Results:** Problematic web-based board gamers showed a lower history of adolescent Internet gaming but a greater rate of problematic Internet gaming compared with healthy web-based board gamers. Moreover, problematic web-based board gamers showed an increase in CES-D, STAI, K-AADHD, and SADS scores but decreased FES scores compared with healthy web-based board gamers. Joblessness; less experience as an Internet gamer; a history of problematic Internet gaming; higher CES-D, STAI, and K-AADHD scores; and lower FES scores were significant predictors of problematic web-based board gaming.

**Discussion:** Psychological, social, and environmental factors can positively influence problematic Web-based board gaming. Healthy Internet gaming during adolescence may play a preventive role in adult problematic web-based board gaming. However, because adolescent problematic Internet gaming tends to lead to problematic web-based board gaming, measures should be taken to prevent it.

## Introduction

Internet gaming is a popular leisure activity worldwide (1). In Korea, 65.7% of the population enjoys Internet gaming, and 90.8% of teenagers play games on the Internet (2). However, concerns about internet gaming are increasing as it becomes more globally popular. Studies have found that gaming can lead to addiction (3–5). The American Psychiatric Association included Internet gaming disorder (IGD) in the Diagnostic and Statistical Manual of Mental Disorders under a provisional status (6), and the World Health Assembly added gaming disorder in the International Classification of Diseases in May 2019 (7).

Several factors have been linked to problematic Internet gaming (8–17). For example, IGD in adolescence is thought to be related to psychological factors, such as mood, anxiety, attention, and impulse control (8–11). Environmental and family factors such as parental monitoring, family conflicts, and family relationships are also considered risk factors for IGD (12, 13).

*Web-based board games* are real-time board games played through online web browsers (18), such as chess, monopoly, backgammon, gomoku, poker, and flower card games (i.e., Korean-style card games). Web-based card games are played with virtual money that can be purchased on the website hosting the game after adult authentication. Players whose daily lives are negatively impacted by these games are referred to as “problematic web-based board gamers” (19, 20). These online casino players show a tendency to chase losses that is greater than that of real-time casino gamblers (21). Internet gambling is illegal in Korea, but some individuals use illegal gambling betting sites employing actual currency, usually through credit cards (22). The terms and conditions of the game prohibit items and accounts from being traded in cash, but some users trade their in-game property for real goods (23).

Problematic web-based board gaming has not been officially designated a formal disorder. Problematic web-based board gaming is thought to have aspects of both Internet gaming disorder and internet-related gambling disorder (20, 24). Moreover, the characteristics of web-based board gaming can be applied to internet-based gambling and illegal online gambling (22). Problematic web-based board game players and individuals with Internet Gaming Disorder (IGD) both tend toward depression, anxiety, and impulsivity (20). In addition, the environmental factors that affect internet-related gambling disorder and internet gaming disorder are both associated with satisfaction with life, well-being, and social adaptation (24). Both Internet gaming and Internet gambling are associated with engagement in reward-seeking behavior without the accomplishment of long-term goals, can cause harm with excessive use, are performed through Internet-enabled devices, and are considered addictive (25–27). However, studies have reported differences between their biological and psychological domains (28–30). Internet gaming can have positive effects on cognitive enhancement and education (28, 29). Individuals with IGD showed increased brain activity within their cognitive network compared with those with Internet-based gambling disorder (30). Despite the ambiguous nosological implications of problematic web-based board gaming, studies of the correlation between IGD in adolescents and problematic web-based board gaming in adults suggest the potential for healthy web-board game play.

Several studies have reported common risk characteristics and transits from problematic Internet gaming to pathologic gambling (31, 32). Problematic Internet use—including in Internet gaming, social media use, web-streaming, pornography viewing, Internet gambling, and buying—has been linked to emotional dysregulation and negative affect (31). The transition from problematic Internet gaming to pathologic gambling has been associated with old age, low self-directedness, and preference for non-strategic gambling (32).

We hypothesized that problematic web-based board gaming behaviors are associated with psychological and social interaction factors. We also hypothesized that problematic adolescent Internet gaming is associated with problematic web-based board gaming in adults.

## Materials and Methods

### Participants

We recruited participants with a special history of web-based board game play by advertising our study online via an online research company as well as offline, including at Chung Ang University and Chung Ang University Hospital from March 2019 to February 2020. People who wanted to participate in the study were invited to visit the IT & Human Research Center at Chung Ang University for screening.

Embrain^®^, a Seoul-based online research company, sent an e-mail to all registered members aged 20 to 60 years. Of these 150,000 members, 4,735 opened the e-mail, and 1,274 completed the screening questions. Of these, 139 satisfied the inclusion criteria and were invited to participate in our study. Of these, 64 accepted the invitation and visited the IT & Human Research Center to participate in the study. Of these 64 people, three were excluded: one due to bipolar disorder, one due to major depressive disorder, and one due to alcohol use disorder. Through banner ads, posters, and flyers at Chung Ang University and Chung Ang University Hospital, 44 people visited the IT & Human Research Center at Chung Ang University. Of the 44 participants, one dropped out due to severe major depressive disorder. Finally, data from 104 participants [61 + 43] were used for the analyses. The inclusion criteria were as follows: (1) age from 20 to 60 years; (2) engagement in web-based board gaming (i.e., flower cards, poker, or Texas holdem) more often than 1 day a week, for a period of more than 1 year, on a legal online site; (3) an official report of web-based board gaming activity, supplied by the web-based board game company at the customer's request; and (4) no history of psychiatric disorders, including substance abuse. The study protocol was approved by the institutional review board of Chung Ang University. Informed consent was obtained from all participants and was confirmed by the board.

### Assessment

#### Demographic Characteristics

The demographic data collected by the study included age, gender, education year, job status, economic status, web-based board game pattern, and history of Internet game play.

#### Pattern of Internet Gaming

The pattern of Internet gaming was assessed using two questionnaires: internet game play time (hour/day) and genre of Internet game. The first question, regarding the participant's Internet game play history, asked, “When you were an adolescent, did you engage in internet gaming at least once a week for 1 year?” The one-week frequency and one-year duration mentioned in the question were based on the IGD research (33) and diagnostic criteria in the DSM-5 (6). The second question, on problematic Internet game play history, asked, “When you were an adolescent, did anyone, important, or close to you consider your gaming to be a problem?” This question has also been used in the IGD research (33).

#### Scales of Psychological Status

We estimated depressive symptoms using the Center for Epidemiologic Studies Depression Scale (CESD). The CESD is a 20-item, four-point self-report instrument (34). The total CESD score ranges from 0 (best) to 60 (worst; 34). A score of 16 is the cut-off point representing “depression” (34). We estimated the presence and severity of symptoms of anxiety, including the propensity to be anxious, using the State-Trait Anxiety Inventory (STAI). The STAI is a 40-item, four-point self-report instrument (35). The total STAI score ranges from 0 (best) to 60 (worst). A score of 30 is the cut-off point representing “anxiety disorder” (35). Attention problems were estimated using the Adult Attention Deficit/Hyperactivity Disorder Self-Report Scale (K-AADHD). The K-AADHD is an 18-item, five-point self-report instrument. The total K-AADHD score ranges from 0 (best) to 72 (worst; 34). The questions in the K-AADHD are split into two parts: a (six questions) and B (12 questions). Four or more positive answers in Part A may indicate ADHD (36, 37).

#### Interaction Scales

Family cohesion was estimated using the Family Environmental Scale (FES). Higher FES scores indicate better family cohesion. The internal consistency of the scale was 0.86 (38, 39). We estimated participants' social intimacy levels using the Social Avoidance and Distress Scale (SADS). The scale consists of 28 questionnaires measuring social anxiety and social avoidance using a five-point Likert scale. The internal consistency of the scale was 0.68 (40, 41).

### Definition of a Problematic Web-Based Board Gamer

We used the Problematic Web Board Gameplay Scale (20) to define problematic web-based board gamers. Those with Problematic Web Board Gameplay Scale scores above 22 (20) and a history of illegal Internet gambling, illegal online money trading with game money within the past month, or purchasing or selling web board identifiers were considered problematic web-based board game players.

### Statistical Analysis

Differences in demographic data between problematic web-based board gamers and healthy web-based board gamers were analyzed using an independent *t*-test or χ2 test. Web-based board gaming patterns and Internet gaming histories were analyzed using independent *t*-tests and the χ2 test. Scores on the psychological and interaction scales were analyzed using independent *t*-tests.

We used hierarchical logistic regression analysis to confirm whether the study's variables could predict statistically significant variance in the dependent variable, for which problematic web-based board gaming was coded as 1, and healthy web-board gaming was coded as 0. Regarding the independent variables, the following discrete set of hierarchical variables was added: demographic factors (age, gender, school year, job, and social economic status) for model 1, model 1 + history of Internet gaming (history of Internet game play and problematic Internet game play) for model 2, model 2 + psychological status (depressed mood, anxiety, attention) for model 3, and model 3 + interaction factors (family environment and social avoidance and distress) for model 4.

The overall fit of each step of the logistic regression model was evaluated with χ2-values (model χ2 and step χ2), while the goodness-of-fit was evaluated with −2 log likelihood. The χ2 values showed the improvement observed in the model, with the predictors relative to the constant-only model or the model preceding the current model. We also evaluated the practical usefulness of each model using tables of classification accuracy to determine the relative success of each model in predicting the correlations with improved golfers. In addition, Nagelkerke's R2 was assessed as an approximate estimate of the amount of variance in the dependent variable accounted for by the model. Wald statistics were used to test whether each individual factor had a significant relationship with improved golfers. When a significant relationship was detected by the Wald test, the interpretation of the coefficient was followed by a determination of the odds ratio—that is, the ratio of the probability that the event (problematic web-based board gaming) would occur to the probability that it would not.

## Results

### Demographic and Web-Based Board Gaming Characteristics

Problematic web-based board gaming was associated with older age (problematic web-based board gamers: 32.9 ± 9.9 vs. healthy web-based board gamers: 27.7 ± 6.4) and joblessness (job/jobless, 26/10 vs. 61/7) compared with healthy web-based board gamers. There were no significant differences in gender, years of education, or socioeconomic status between the two groups (see Table 1).

**Table 1:** Demographic and gaming characteristics of web-based board gamers.

	**Healthy gamers (*n* = 68)**	**Problematic gamers (*n* = 36)**	**Statistics**
**Demographic data**			
Age (years)	27.7 ± 6.4	32.9 ± 9.9	*t* = −3.27, *p* < 0.01^*^
Gender (man/woman)	38/30	18/18	χ^2^ = 0.33, *p* = 0.68
Education (year)	14.5 ± 1.6	15.0 ± 1.8	*t* = −1.41, *p* = 0.16
Job status (yes/no)	61/7	26/10	χ^2^ = 5.26, *p* = 0.03^*^
**Socioeconomic status ($/year)**			
< $20,000	10	10	χ^2^ = 3.19, *p* = 0.20
$20,000–40,000	50	24	
> $40,000	8	2	
**Web-based board gaming pattern**			
Problematic web board game play scale	17.9 ± 4.3	26.6 ± 3.9	*t* = −10.1, *p* < 0.01^*^
Number of logins per day	1.3 ± 1.5	2.2 ± 1.8	*t* = −2.51, *p* = 0.01^*^
Play time (hour/day)	1.3 ± 0.6	1.6 ± 0.8	*t* = −2.06, *p* = 0.04^*^
Winning rate (%)	40.4 ± 11.4	42.7 ± 9.5	*t* = −1.04, *p* = 0.30
**Internet game play history**			
History of Internet game play (yes/no)	41/27	14/22	χ^2^ = 4.33, *p* = 0.04^*^
Problematic Internet game play (yes/no)	4/64	12/24	χ^2^ = 13.63, *p* < 0.01^*^
Play time (hour/day)	1.5 ± 0.6	3.8 ± 1.9	*t* = −6.98, *p* < 0.01^*^
**Genre of internet game**			
MMORPG	16	6	χ^2^ = 0.43, *p* = 0.93
RTS	15	4	
FPS	6	2	
Others	4	2	

* *Statistically significant*.

Problematic web-based board gamers showed higher Problematic Web Board Gameplay Scale scores (26.6 ± 3.9 vs. 17.9 ± 4.3), number of logins (2.2 ± 1.8 vs. 1.3 ± 1.5), and play time (1.6 ± 0.8 vs. 1.3 ± 0.6 hours/day) compared with healthy web-based board gamers. There was no significant difference in the game-winning rate between the two groups (see Table 1).

**Figure 1:**
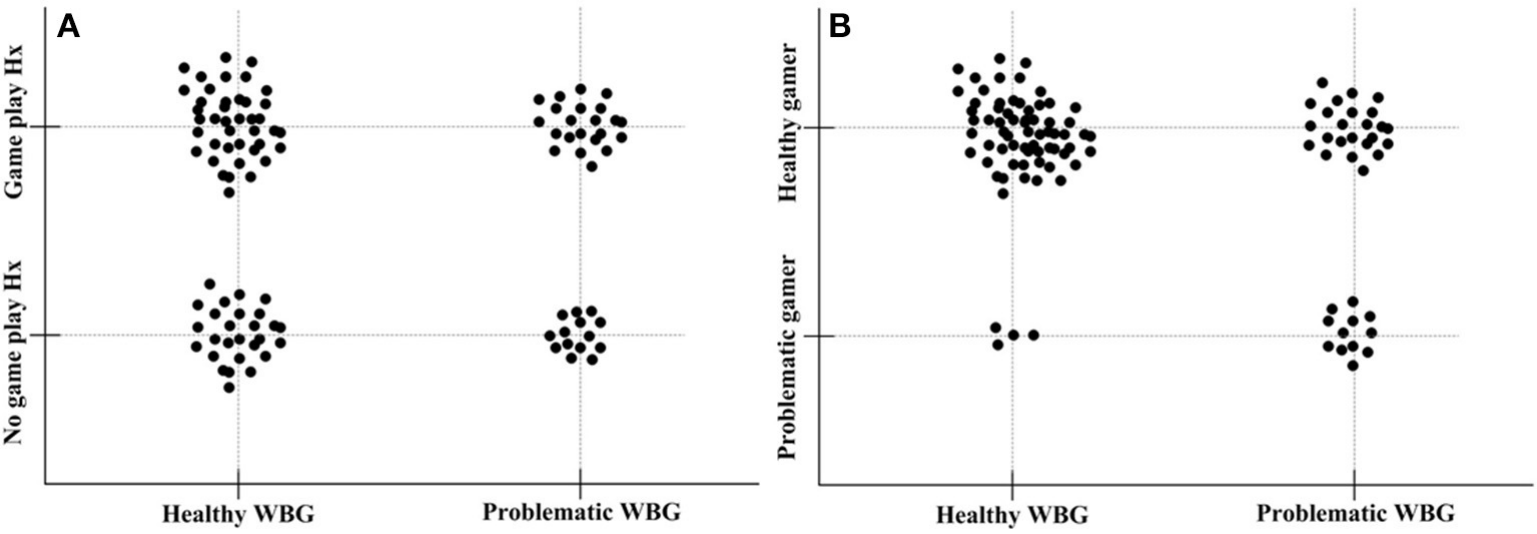
The correlation between internet gaming and web-based board gaming. **(A)** The correlation between history of internet game play (Game play Hx) and problematic web-based board game play (Problematic WBG). **(B)** The correlation between history of problematic internet game play (Problematic gamer) and problematic web-based board game play (Problematic WBG).

Problematic web-based board gamers showed a shorter history of adolescent Internet gaming (yes/no, 14/22 vs. 41/27) but a higher rate of problematic Internet gaming (yes/no, 12/24 vs. 4/64) compared with healthy web-based board gamers (see Table 1 and Figure 1). In addition, problematic web-based board gamers showed longer Internet game play time compared to healthy web-based board gamers. There was no significant difference in game genre between the two groups.

### Psychological and Social Interaction Scale

Problematic web-based board gamers showed higher scores on the CES-D (problematic web-based board gamers: 15.3 ± 11.1 vs. healthy web-based board gamers: 6.4 ± 6.3), STAI (85.9 ± 19.9 vs. 76.5 ± 16.5), and K-AADHD (15.6 ± 7.5 vs. 6.6 ± 5.1) scores compared with healthy web-based board gamers (see Table 2). Problematic web-based board gamers showed lower scores on the FES (27.1 ± 5.4 vs. 34.9 ± 4.7) but higher SADS (80.3 ± 20.1 vs. 72.3 ± 16.9) scores compared with healthy web-based board gamers (Table 2).

**Table 2:** Comparison of scores on the psychological and social interaction scales.

	**Healthy gamers (*n* = 68)**	**Problematic gamers (*n* = 36)**	**Statistics**
**Psychological scale**			
CES-D	6.4 ± 6.3	15.3 ± 11.1	*t* = −5.24, *p* < 0.01^*^
STAI	76.5 ± 16.5	85.9 ± 19.9	*t* = −2.55, *p* = 0.01^*^
K-AADHD	6.6 ± 5.1	15.6 ± 7.5	*t* = −7.21, *p* < 0.01^*^
**Interaction scale**			
FES	34.9 ± 4.7	27.1 ± 5.4	*t* = 7.62, *p* < 0.01^*^
SADS	72.3 ± 16.9	80.3 ± 20.1	*t* = −2.14, *p* = 0.04^*^

*CES-D, Center for Epidemiologic Studies Depression Scale; STAI, state-trait anxiety inventory; K-AADHD, adult attention deficit/hyperactivity disorder scale; FES, family environment scale; SADS, social avoidance and distress scale*.

### Hierarchical Logistic Regression Analysis

A Durbin–Watson test indicated that there was no autocorrelation in the data. Of the four models employed, all were significantly associated with problematic web-based board gaming. Considering its highest step χ2 value and the improved classification accuracy, adolescent Internet gaming history was the strongest factor for problematic web-based board gaming among all the domains. Considering its highest model χ2 value and the improved classification accuracy, Model 4, which included all domains, predicted problematic web-based board gaming most strongly among the models.

In Model 1, the χ2 (21.9, *p* < 0.01) and Nagelkerke's R2 (0.262, 26.2% variance in the dependent variable of problematic web-based board gaming) indicated that the model was good enough to predict problematic web-based board gaming. When we examined the practical usefulness of the model based on classification accuracy, five variables in Model 1 enhanced the prediction accuracy of the group membership of the dependent variable to 73.1%. The step χ2 value (step χ2 = 21.9, *p* < 0.01) showed that demographic factors were the main predictive factors for problematic web-based board gaming. In Model 2, the χ2 (56.4, *p* < 0.01) and Nagelkerke's R2 (0.578, 57.8% variance in the dependent variable of problematic web-based board gaming) indicated that the model was good enough to predict problematic web-based board gaming. The seven variables in Model 2 enhanced the prediction accuracy of the group membership of the dependent variable by 84.8%. The step χ2 value (step χ2 = 34.5, *p* < 0.01) showed that the factors of pattern of internet game play were the main predictive factors for problematic web-based board gaming. In Model 3, the χ2 (77.0, *p* < 0.01) and Nagelkerke's R2 (0.722, 72.2% variance in the dependent variable of problematic web-based board gaming) indicated that the model was good enough to predict problematic web-based board gaming. Ten variables in Model 3 enhanced the prediction accuracy of the group membership of the dependent variable to 89.4%. The step χ2 value (step χ2 = 20.7, *p* < 0.01) showed that the factors of psychological status were the main predictive factors for problematic web-based board gaming. In Model 4, the χ2 (94.1, *p* < 0.01) and Nagelkerke's R2 (0.821, 82.1% variance in the dependent variable of problematic web-based board gaming) indicated that the model was good enough to predict problematic web-based board gaming. The 12 variables in Model 4 enhanced the prediction accuracy of the group membership of the dependent variable to 94.14%. The step χ2 value (step χ2 = 16.9, *p* < 0.01) showed that the interaction factors were the main predictive factors for problematic web-based board gaming (see Table 3).

**Table 3:** Results of the hierarchical logistic regression analysis.

**Independent variable**		**Model 1**			**Model 2**			**Model 3**			**Model 4**		
		**Beta**	**Wald**	**OR**	**Beta**	**Wald**	**OR**	**Beta**	**Wald**	**OR**	**Beta**	**Wald**	**OR**
Demographic	Age	0.084	7.524	1.088^**^	0.159	11.637	1.172^*^	0.141	7.695	1.151^**^	0.111	3.328	1.118
factors	Gender	0.068	0.018	1.070	0.138	0.048	1.148	0.269	0.092	1.309	2.099	2.294	8.158
	School	0.051	0.103	1.052	−0.315	2.363	0.730	−0.520	3.341	0.594	−0.801	2.967	0.449
	Job	−1.598	6.101	0.202^*^	−1.719	4.539	0.179^*^	−1.779	2.968	0.169	−2.917	3.900	0.054^*^
	SES		7.374			5.003			0.919			0.154	
	SES (1)	2.704	5.134	14.944	2.738	4.937	15.463	0.453	0.070	1.574	−0.459	0.051	0.632
	SES (2)	1.336	1.441	3.804	2.332	3.767	10.296	1.252	0.678	3.498	−0.769	0.150	0.463
Pattern of	Hx Game				−5.572	17.042	0.004^**^	−5.944	10.618	0.003^**^	−3.681	2.255	0.025^*^
Internet game play	P Game				3.703	11.564	40.583	4.258	7.372	70.659	6.296	5.921	542.627^**^
Psychological	CES-D							0.134	3.538	1.143	0.237	4.514	1.268^*^
status	STAI							−0.070	3.631	0.933	−0.130	5.359	0.878^*^
	K-AADHD							0.236	8.747	1.266^**^	0.177	4.561	1.194^*^
Interaction	FES										−0.553	6.174	0.575^*^
factors	SADS										0.073	3.372	1.075
**Indices**	**Model 0**	**Model 1**			**Model 2**			**Model 3**			**Model 4**		
−2LL	134.14	112.22			77.76			57.11			40.11		
Step χ^2^/p	N/A	21.9/<0.01			34.5/<0.01			20.7/<0.01			16.9/<0.01		
Model χ^2^/p	N/A	21.9/<0.01			56.4/<0.01			77.0/<0.01			94.1/<0.01		
Nag 2	N/A	0.262			0.578			0.722			0.821		
Class Accur	65.4	73.1			84.8			89.4			94.1		

* *p < 0.05*,

** *p < 0.01;−2LL,−2 log likelihood; Nag R2, Nagelkerke's R2; class accur, classification accuracy; dependent factor, problematic web-based board game play; SES, social economic status; Hx game, history of internet game play; P game, problematic internet game play; CES-D, Center for Epidemiologic Studies Depression Scale; STAI, State-Trait Anxiety Inventory; K-AADHD, adult attention deficit/hyperactivity disorder scale; FES, family environment scale; SADS, social avoidance and distress scale*.

The results of the Wald's statistics for all independent variables indicated that joblessness; less experience of Internet gaming; problematic Internet gaming history; higher scores on the CES-D, STAI, and K-AADHD; and lower scores on the FES were significant predictors of problematic web-based board gaming (see Table 3).

## Discussion

Problematic web-based board gaming was associated with a shorter history of adolescent Internet gaming but a greater rate of problematic Internet gaming compared with healthy web-based board gamers. Problematic web-based board gaming was associated with higher CES-D, STAI, K-AADHD, and SADS scores but lower FES scores than healthy web-based board gaming. Overall, joblessness; less experience with Internet gaming; a history of problematic Internet gaming; higher scores on the CES-D, STAI, and K-AADHD; and lower FES scores were significant predictors of problematic web-based board gaming.

Considering the step χ^2^ values, this study found that demographic factors could be significant predictive factors for problematic web-based board game play. Of the demographic domains, such as IGD (35, 36), problematic web-based board gaming was associated with joblessness. Young asserted that problematic Internet use could aggravate occupational impairment (42). Kim et al. reported that adults with IGD are more likely to be unemployed than healthy individuals (43).

Of the four domains, Internet gaming pattern was found to be the most crucial for problematic web-based board gaming. Considering the negative beta value of Internet game play history, experience of adolescent Internet gaming would be negatively correlated with adult problematic web-based board gaming. Considering its positive beta value, history of problematic adolescent Internet gaming was associated with adult problematic web-based board gaming. The beta value of the regression analysis was reflected in the slope of the regression line (44). Taken together, the two results concerning the Internet gaming pattern domain suggest that individuals with a history of Internet gaming but no history of problematic Internet gaming do not have a higher probability of problematic web-based board gaming.

These results offer new insights into the prevention of problematic web-based board gaming. In contrast to our results, several previous studies have demonstrated the risks of early exposure to Internet gaming or gambling (42–46). The results of retrospective studies by Shaffer et al. (47) and Abbott et al. (48) indicated that adults who are problematic gamblers are likely to have gambled in their adolescence and that the younger they are when exposed to gambling, the more likely they are to experience subsequent problems related to it (49). Similarly, Ni et al. (50) reported that the age at first exposure to Internet gaming was associated with Internet addiction. However, our results reported that the experience of adolescent Internet gaming did not lead to problematic web-based board gaming in adults, but served as a protective factor. Caretakers' care for and interest in their child's Internet gaming pattern and Internet use are thought to be important factors for Internet gaming disorder (12, 13). Kwak et al. (51) compared the changes in behavioral patterns and brain activities between problematic gaming students and student pro-gamers for 1 year. Despite their heavy exposure to Internet gaming, student pro-gamers, who had planned gaming schedules and gaming discipline, showed fewer problematic behaviors than problematic gaming students did. Jones et al. found that a moderate level of gaming may positively influence well-being by improving mood, regulating emotions, and reducing stress (52). The authors also suggested that relationships with peers and socializing with other players promoted positive social functioning. Hence, support from parents and teachers can prevent problematic online gaming (12, 46). The protective effect found in our study might be associated with adaptation to the online environment, which may protect against problematic web-based board gaming.

Several studies have shown that, for complicated reasons, a problematic Internet gaming history could be a higher risk for problematic Internet gaming or gambling than exposure to Internet gaming or gambling itself (43, 45). Karlsson et al. (53) reported that problem gaming and problematic Internet use are associated with problem gambling. Problematic Internet gaming and problematic gambling share similar risk factors, including male gender, social isolation, feelings of loneliness, and underlying psychiatric diseases such as attention deficit hyperactivity disorder and major depressive disorder (43, 45, 54, 55).

We have already shown that problematic web-based board gaming may be linked to users' psychological and social problems (24). Similarly, in this study, problematic web-based board gamers showed more depression, anxiety, and attention deficiency as measured with psychological scales, along with less family cohesion and social intimacy as measured with interaction scales. Multiple studies have suggested that psychological and environmental factors are strongly correlated with IGD (12–17). Specifically, parental care (12) and teacher support can enhance social engagement, which can help prevent IGD (46).

## Limitations

This study has several limitations. First, the sample size was relatively small and had a cross-sectional design; therefore, the results may have limited generalizability. Future studies should consider a longitudinal design with a larger population. Second, we did not examine adolescent web-based board gaming history or adult Internet gaming history. There may be correlations between these two factors; however, this study recruited only web-based board gamers and collected data on their Internet gaming. Thus, further studies are required to classify the characteristics of each group. Finally, the study did not assess tobacco information details. Tobacco habits are known to be associated with gambling disorder. Future studies should assess the relationship between tobacco habits and web-based board gameplay.

## Conclusion

The results of this study showed that psychological, social, and environmental factors can positively influence problematic web-based board gaming. Healthy Internet gaming during adolescence may play a preventive role against problematic web-based board gaming during adulthood. However, measures should be taken to prevent problematic adolescent Internet gaming because it tends to lead to problematic web-based board gaming.

## Data Availability Statement

The raw data supporting the conclusions of this article will be made available by the authors, without undue reservation.

## Ethics Statement

The studies involving human participants were reviewed and approved by Institutional Review Board of the Chung Ang University. The patients/participants provided their written informed consent to participate in this study.

## Author Contributions

SB and DH contributed to the conception of the study. DH and KM contributed to the study methodology. SK contributed to the formal analysis of the study. HR contributed to the investigation of this study, preparation of the original draft, review, and editing of the manuscript. DH supervised the study. All authors approved the final manuscript as submitted and agree to be accountable for all aspects of the work.

## Conflict of Interest

The authors declare that the research was conducted in the absence of any commercial or financial relationships that could be construed as a potential conflict of interest.
